# The course of knee extensor strength after total knee arthroplasty: a systematic review with meta-analysis and -regression

**DOI:** 10.1007/s00402-022-04750-5

**Published:** 2023-01-13

**Authors:** Ravi Singla, Daniel Niederer, Alexander Franz, Kevin Happ, Christoph Zilkens, Patrick Wahl, Michael Behringer

**Affiliations:** 1 Department of Orthopedics and Trauma Surgery, University Hospital Bonn, Bonn, Germany; 2 Department of Adult Reconstruction, ATOS Orthoparc Clinic Cologne, Cologne, Germany; 3 Institute of Cardiology and Sports Medicine, German Sport University Cologne, Cologne, Germany; 4 Department of Sports Medicine and Exercise Physiology, Institute of Occupational, Social and Environmental Medicine, Goethe University Frankfurt, Frankfurt, Germany; 5 Department of Sports Sciences, Goethe University Frankfurt, Frankfurt, Germany

**Keywords:** Total knee replacement, Total knee arthroplasty, Quadriceps femoris, Muscle, Strength, Contralateral leg

## Abstract

**Purpose:**

Muscular strength loss and atrophy are postoperative complications. This systematic review with meta-analysis investigated the course of on knee extensor mass and strength from pre-surgery over total knee arthroplasty to rehabilitation and recovery.

**Methods:**

A systematic literature search was conducted in PubMed (Medline), Cochrane Library (CINAHL, Embase) and Web of Science (until 29th of June 2022). Main inclusion criteria were  ≥ 1 preoperative and  ≥ 1 measurement  ≥ 3-months post-operation and ≥ 1 objective assessment of quadriceps strength, muscle mass or neuromuscular activity, measured at both legs. Studies were excluded if they met the following criteria: further impairment of treated extremity or of the contralateral extremity; further muscle affecting disease, or muscle- or rehabilitation-specific intervention. The Robins-I tool for non-randomized studies, and the Cochrane Rob 2 tool for randomized controlled studies were used for risk of bias rating. Pre-surgery, 3 months, 6 months and 1 year after surgery data were pooled using random effects meta-analyses (standardized mean differences, SMD, Hedge’s g) in contrast to the pre-injury values.

**Results:**

1417 studies were screened, 21 studies on 647 participants were included. Thereof, 13 were non-randomized controlled trails (moderate overall risk of bias in most studies) and 7 were randomized controlled trials (high risk of bias in at least one domain in most studies). Three (*k* = 12 studies; SMD = − 0.21 [95% confidence interval = − 0.36 to − 0.05], *I*^2^ = 4.75%) and six (*k* = 9; SMD = − 0.10 [− 0.28 to − 0.08]; *I*^2^ = 0%) months after total knee arthroplasty, a deterioration in the strength of the operated leg compared with the strength of the non-operated leg was observed. One year after surgery, the operated leg was stronger in all studies compared to the preoperative values. However, this increase in strength was not significant compared to the non-operated leg (*k* = 6, SMD = 0.18 [− 0.18 to 0.54], *I*^2^ = 77.56%).

**Conclusion:**

We found moderate certainty evidence that deficits in muscle strength of the knee extensors persist and progress until 3 months post-total knee arthroplasty in patients with end-stage knee osteoarthritis. Very low certainty evidence exists that preoperatively existing imbalance of muscle strength and mass in favor of the leg not undergoing surgery is not recovered within 1 year after surgery.

## Introduction

Osteoarthritis (OA) of the knee is associated with decreased functional activity, progressive knee pain and severe immobilization-induced skeletal muscle atrophy [10]. The surgical restoration of joint function by total knee arthroplasty (TKA) is the last option to decrease subjective pain and enhance quality of life in concerned patients.

The rehabilitation after TKA is often accompanied by long-term deficits in skeletal muscle health (SMH) such as muscle atrophy, strength losses and impaired neuromuscular activity [13]. These deficits are most often already present preoperatively [10] and exacerbate during the surgical procedure and subsequent hospitalization [32]. Although state-of-the art rehabilitation concepts try to reduce postoperative declines in SMH and support joint function, muscular deficits continue to develop progressively and can be demonstrated even years after TKA [24, 47]. Since it is known that preoperative muscle strength is associated with good performance outcomes after TKA [34, 58], the importance of skeletal muscle health for the success of the post-operative therapy is given.

Knowing the course of muscle strength in a standard care population would be helpful to rate the effectiveness of interventional trials on pre- and postoperative rehabilitation measures in TKA and, from a practical point-of-view, to identify patients with a below-mean deficit; these could be treated more deficit-orientated.

Since the usual course of muscle mass and strength following surgical therapy and subsequent standard rehabilitation is unknown, this systematic review with meta-analysis investigated the course of on knee extensor mass and strength from pre-surgery over total knee arthroplasty to rehabilitation and recovery.

## Methods

### Inclusion criteria

Studies were eligible for inclusion if they met the following criteria: (a) measurement of the quadriceps femoris muscle strength or measurement of muscle mass or measurement of neuromuscular activity; (b) measurement in both legs; (c) standardized measurement technique (d) study text available in German or English, (e) preoperative values of at least one outcome of interest reported; (f) follow-up measurements at least 3 months post-surgery; (g) maximum effort quadriceps strength measurement; (h) randomized controlled trial or cohort study.

### Exclusion criteria

Studies were excluded if they met following criteria: (a) further or secondary impairment(s) of the treated extremity; (b) additional impairment of the contralateral extremity; (c) further muscle affecting disease (neurological, rheumatological, oncological); (d) muscle- or rehabilitation- specific interventions beyond the medically prescribed formal standard care/rehabilitation (such as exercise interventions as part of a therapy RCT). (e) strength values taken from questionnaires; (f) strength values just measuring angle to extension maximal force momentum.

### Information sources

The following databases were used for searching: The Cochrane library (CENTRAL, including EMBASE and CINAHL), Web of Science, and PubMed; from January 2000 to June 2022. From the studies included, all reference lists were screened for further eligible studies.

### Search strategy

To find appropriate studies the following eight searching combinations, with the database-specific Boolean operators, were entered in each database: 1. “total knee arthroplasty” AND “muscle mass” 2. “total knee arthroplasty” AND “strength” 3. “total knee arthroplasty” AND “neuromuscular activation” 4. “total knee arthroplasty” AND “contralateral leg” 5. “total knee replacement” AND “muscle mass” 6. “total knee replacement” AND “strength” 7. “total knee replacement „AND “neuromuscular activation” 8. “total knee replacement” AND “contralateral leg”.

### Selection process

Each step of the selection process was done by two independent examiners (RS, AF). The comparison of the studies was done at the full-text-retrieval step. Disparities in the included studies were discussed; a third reviewer (MB) was included if no consent could be found.

**Fig. 1:**
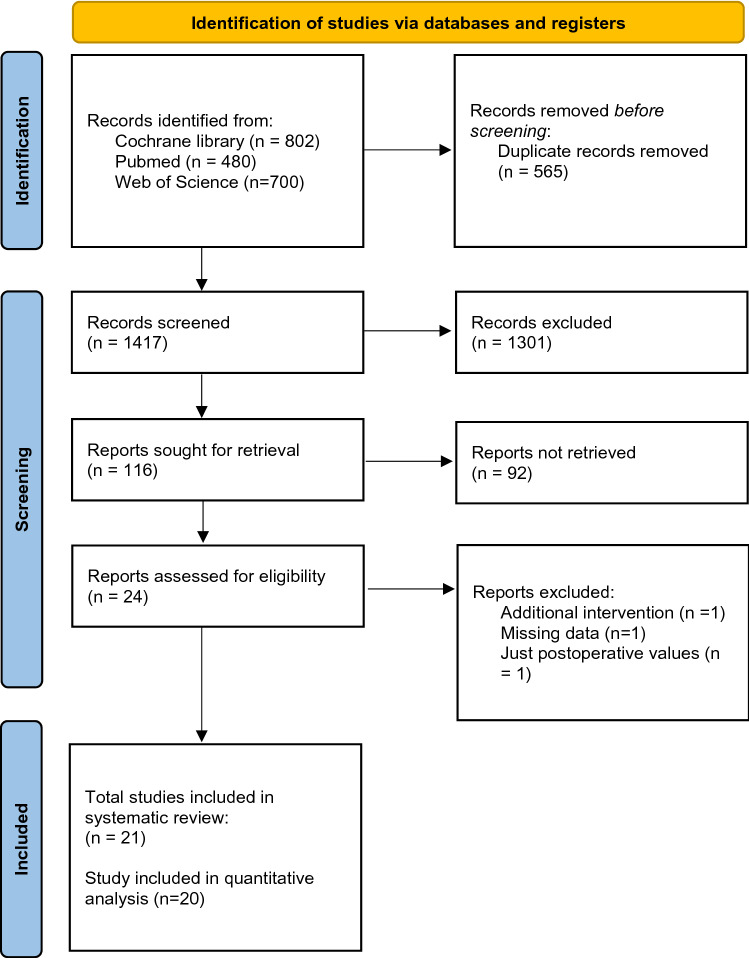
PRISMA 2020 flow diagram for new systematic reviews including searches of databases, registers and other sources for the present study

Selection of studies is shown in the PRISMA Flow Chart (Fig. 1). After deleting duplicate articles, the titles were assessed according to the previously determined inclusion and exclusion criteria. Afterwards the examiners analyzed the abstracts of the studies. The full texts for the abstract considered eligible were afterwards retrieved. If studies were not obtainable, the corresponding authors were contacted via email. Data were extracted from the full text of studies included in the systematic review. Duplicate removal and comparison between the two reviewers was done using Microsoft Excel (Microsoft Corporation, Version 16.47.1).

### Data collection process

Data collection was done by the same two reviewers using the same procedure as during the selection process. Both researchers read all studies and extracted all possible data into one overview table, in Excel (Microsoft Corporation, Version 16.47.1), each. After data extraction both overview tables were compared and merged.

### Data items

At the beginning of the data extraction following data were collected and summarized in an Excel table (Microsoft Corporation, Version 16.47.1):

- General information (number of participants, patient characteristics, study characteristics).
- Outcomes (strength measurements, muscle mass measurements, muscle activation, wellbeing, range of motion and performance measurements).

The (outcomes, dependent variables) data that had been extracted in addition to strength, muscle mass and neuromuscular activity were analyzed for potential subgroup analyses and meta regressions. For inclusion in the meta-analyses parameters had to be obtained with a standardized measurement tool, adequate techniques and analysis of both legs had to be done and taken at least in three studies at the same time point.

### Study risk of Bias assessment

The Robins-I tool [43] was used to assess controlled non-randomized before and after studies. The Risk of Bias (RoB) 2 tool [44] was applied for randomized trials. Risk of Bias was rated by two independent researchers. After assessing all studies (i.e., outcomes) differences were resolved by discussion. Only data that were relevant for the systematic review were extracted from the individual studies.

**Fig. 2:**
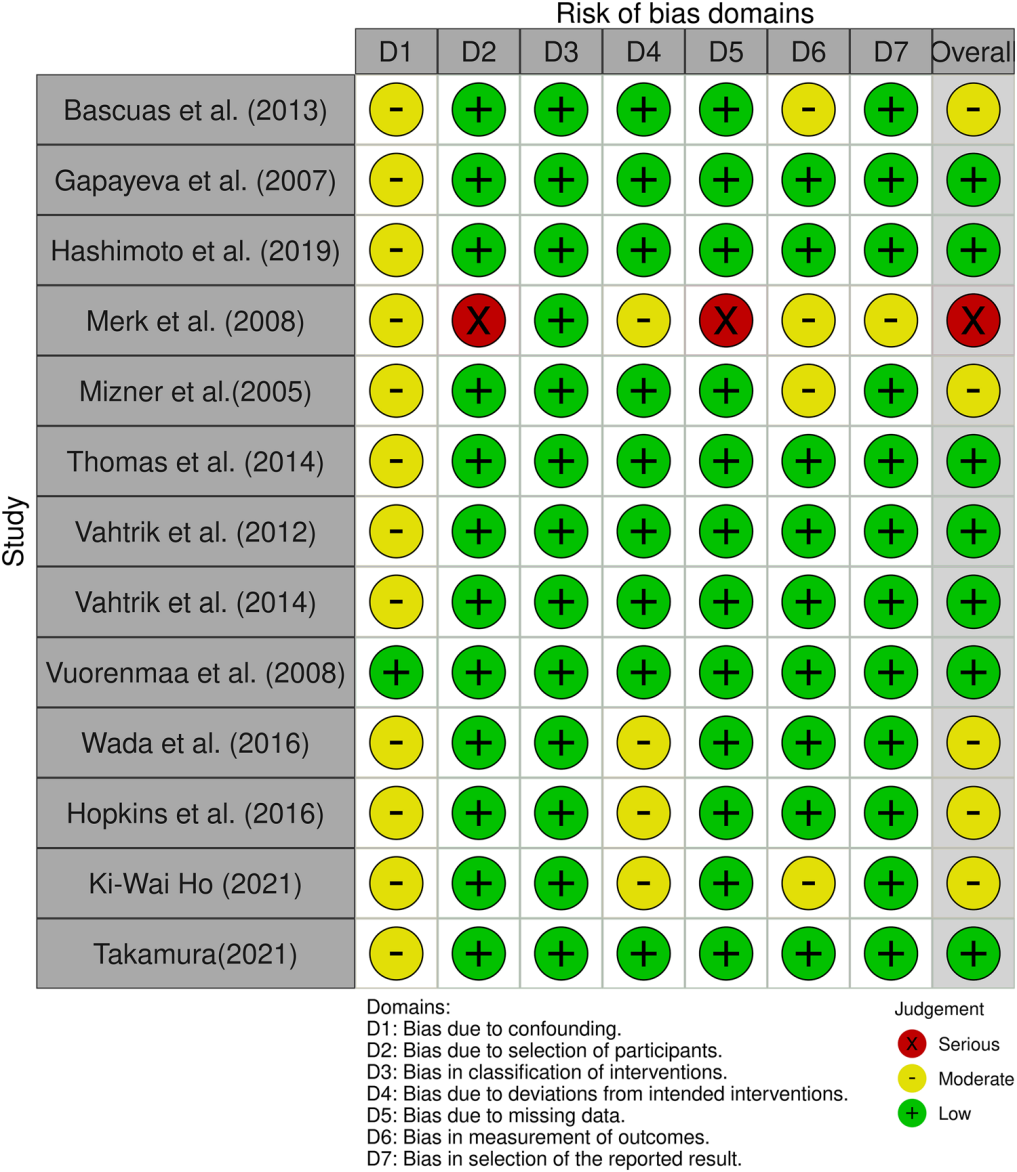
“Traffic light” plots of the study judgements using Robins-I

**Fig. 3:**
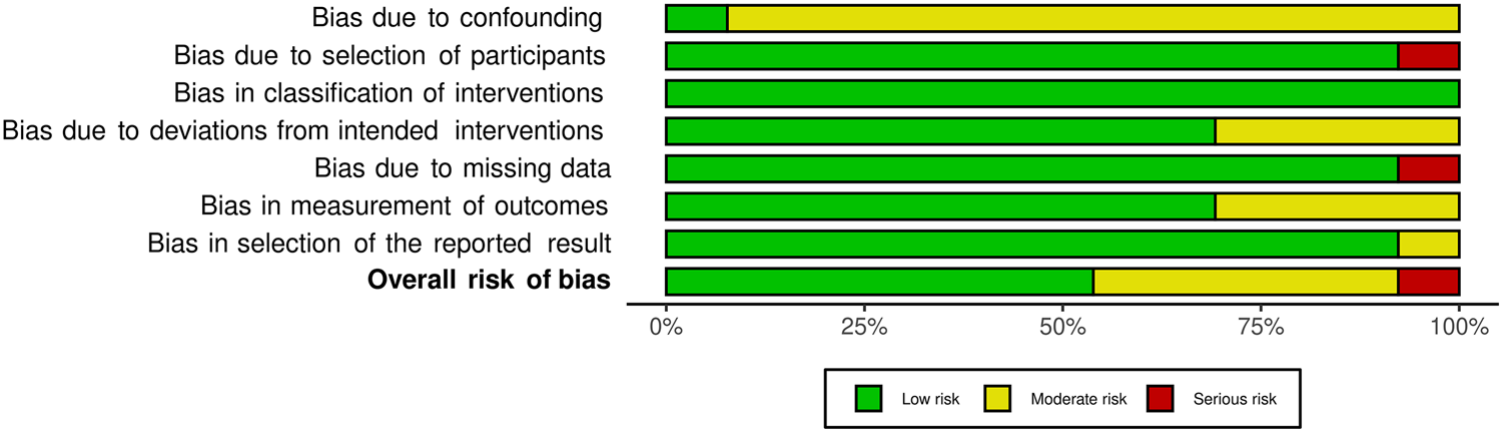
Weighted bar plots of the distribution of risk-of-bias judgements in each bias domain of all outcomes assessed in studies judged with Robins-I

**Fig. 4:**
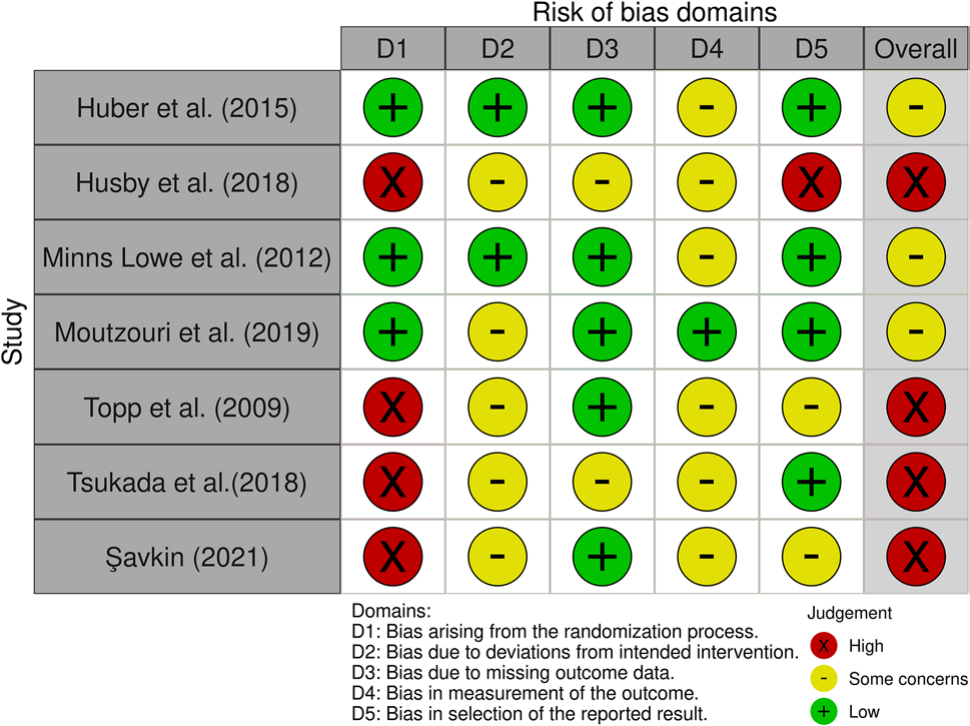
“Traffic light” plots of the study judgements using RoB 2

**Fig. 5:**
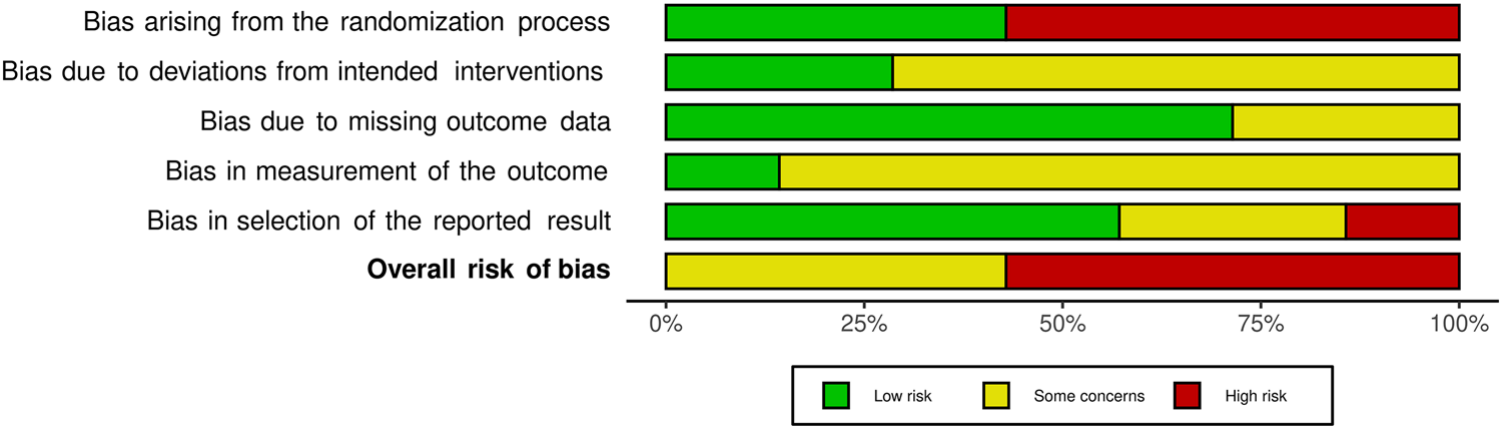
Weighted bar plots of the distribution of risk-of-bias judgements in each bias domain of all outcomes of the studies judged with RoB

Results from both analyses, Robins I and RoB 2, were converted from an Excel table (Microsoft Corporation, Version 16.54) with the robvis visualization tool [28] and displayed as a traffic light and as summary plots (Figs. 2, 3, 4 and 5).

### Effect measures

Effect sizes for each outcome were calculated as standardized mean differences (SMD, pre- to post-surgery values, always in comparison to the contralateral control leg) in the form of Hedge’s g. For all outcomes with preoperative and postoperative values, for operated and non-operated leg, effect sizes were calculated.

### Synthesis method

Measurements were selected at the closest time points before injury and 3 months, 6 months, or 1 year after surgery. For each of these timepoints, a pairwise (in comparison to the contralateral value) meta-analysis in comparison to the pre-surgery strength was calculated.

If mean and standard deviation could not directly be retrieved from the original study, data were imputed following the recommendations made by the Cochrane collaboration.

### Main analyses

Effect size and its variance were used to perform forest plots and heterogeneity analyses in Jamovi (Version 1.8.2.0, jamovi.org, Sydney, Australia) using the module “Major”. The meta-analyses were performed with random effects models. Weighted standardized mean differences in the form of Hedge´s g were used as effect size estimators. Mean effect sizes and meta-analyses estimates (95% confidence interval, *p* value) were calculated for the analyses. *Z*-Statistics at a 5% alpha-error-probability were calculated to test for overall effects.

For the calculation of the between effects heterogeneity, Tau^2^, the maximum restricted likelihood approach was used. Besides, *I*^2^ was calculated for the between effects heterogeneity.

### Multilevel meta-regression

A multilevel meta-regression was done in “R” (R Core Team, Version 1.4.1106, Vienna, Austria) to investigate whether the effect sizes correlate with the following independent moderators: Age, Body Mass Index (BMI), time after the operation (months) and the share of female participants. Meta-regression estimates, its standard error and the 95% confidence interval were calculated. Since the independent variables do not exhibit within-study variability, tau^2^ could not be calculated. Therefore, (Cochranes) *Q* was calculated to determine heterogeneity of the results. *R*^2^ was calculated to calculate how much of the heterogeneity in the meta-analyses can be explained by the moderators.

### Sensitivity analysis

A sensitivity analysis was not conducted because all three meta-analyses consisted of an insufficient number of studies.

### Reporting Bias assessment

Funnel plots were plotted and Egger’s regression tests to detect any funnel asymmetry were performed for reporting bias assessment. For both, Jamovi (The jamovi project (2021). jamovi (Version 1.6) [Computer Software]. Retrieved from https://www.jamovi.org) with the Modul “Major” was used. Reporting bias assessment was just reported for the 3 months analysis because it was the only meta-analysis consisting of ten studies [42].

### Certainty assessment

The quality of evidence in each meta-analysis was evaluated with the “Grades of Research, Assessment, Development and Evaluation” (GRADE) approach. The evidence derived by the meta-analyses was classified in “high”, “moderate”, “low” or “very low”. Each certainty of evidence could then be up-or downgraded based on the following five criteria: 1. Risk of Bias; 2. Inconsistency; 3. Indirectness; 4. Imprecision; 5. Publication Bias.

## Results

### Study selection

The study flow is depicted in Fig. 1. Of the 24 studies assessed for eligibility, one was excluded because patients underwent pre-operative rehabilitation [53] and one study specified a strength measurement but just angle-to-extension maximal force momentum was reported [2]. One study [23] measured muscle mass only postoperatively. Finally, 21 studies were included (Fig. 1). 20 studies reported bilateral strength measurements and were included in the meta-analyses. One study measured bilateral leg lean tissue mass and was included in the systematic review but not in the meta-analyses [19].

### Study characteristics

Detailed study characteristics are shown in Table 1. Moutzouri et al. [36] measured the strength values after fourteen weeks. These results were included in the meta-analysis 3 months after the surgery. Merk et al. [29] measured strength 5–7 months post-operatively. Those results were included in the 6 months postoperative analysis. Unlike other studies, Merk et al. [29] differentiated between contralateral leg with and without previous total knee replacement. In this review strength values from the contralateral leg without previous knee arthroplasty was used for meta-analyses.

**Table 1:** Overview of general information and outcomes

Subject data				Measurement variable							
Authors	Participants (female/male)	Preoperative age	Preoperative BMI (kg/m^2^)	Measurement points	Strength operated leg	Strength non-operated leg	Preoperative contralateral strength difference (%) (mean strength operated leg/mean strength non-operated leg*100)	Force measurement (unit)	Force measuring device	Data extraction	Country
Bascuas et al. [3]	44 (32/12)	71.4 ± 7.12	32.65 ± 5.02	Preoperative One year postoperative	21,28 ± 8,22 23,00 ± 7,61	21,57 ± 7,67 25,83 ± 10,72	98,66	Max. isometric muscle strength (kg)	Isometric dynamometry	Median = mean; IQR in SD	Spain
Gapeyeva et al. [14]	10 (10/0)	63 ± 5.5	29 ± 4	Preoperative Six months postoperative	159,45 ± 47,91 173,16 ± 38,80	233,04 ± 57,05 252,53 ± 52,49	68,42	Max. isometric voluntary contraction (N)	Chair fixed dynamometer	WebPlotDigizier, SE in SD	Estonia
Hashimoto et al. [17]	83 (83/0)	75.6 ± 7.2	25.4	Preoperative One year postoperative	0,29 ± 0,11 0,37 ± 0,11	0,33 ± 0,11 0,38 ± 0,12	87,88	Max. isometric contraction (kgf/kg)	Handheld dynamometer mTas F1	SE in SD	Japan
Huber et al. [20]	23 (10/13)	71.9 ± 8.1	30.8 ± 4.9	Preoperative Three months postoperative	247,60 ± 100,50 204,10	315,40 ± 123,1 279,10	78,50	Isometric muscle strength (N)	Handheld pull gauge	Mean derived from change of first measurement	Swizerland
Husby et al. [22]	20 (12/8)	63	28.6 ± 5.1	Preoperative One year postoperative	18,20 ± 8,50 20,57	22,50 ± 9,4 30,15	80,89	1 RM (kg)	Leg extensor press	Mean derived from change of first measurement	Norway
Lowe et al. [30]	51 (30/21)	70.76 ± 9.45	29.27 ± 5.82	Preoperative Three months postoperative One year postoperative	0,34 ± 0,42 0,72 ± 0,50 0,87 ± 0,79	0,57 ± 0,50 0,88 ± 0,64 0,83 ± 0,76	59,65	Leg extensor Power (W/kg)	Leg extensor press	Median = mean; IQR in SD	United Kingdom
Merk et al. [29]	53 (43/10)	64.68 ± 8.56	28.57 ± 2.09	Preoperative three months Postoperative Five-seven months postoperative	39,00 ± 21,73 42,70 ± 22,09 55,20 ± 29,34	76,3 ± 39,87 81,3 ± 40,8 83,9 ± 41,03	51,11	Max. isokinetic muscle strength (60°/sec.) (Nm)	Isokinetic MessSystem Cybex 340	Median = mean; confidence interval in SD	Germany
Mizner et al. [31–33]	40 (18/22)	64 ± 9	31.4 ± 3.7	Preoperative Three months postoperative Six months postoperative	18, 00 ± 8,00 15,00 ± 6,00 18,00 ± 8,00	23,00 ± 10,00 23,00 ± 10,00 23,00 ± 10,00	78,26	Max. isometric muscle strength (N/BMI)	Electromechanical dynamometer	Everything was given as needed	United States
Moutzouri et al. [36]	25	72.3 ± 5.6		Preoperative 14 weeks postoperative	41,80 ± 17,80 55,40 ± 23,50	57,20 ± 16,30 69,10 ± 24,40	73,08	Isokinetik knee extensors’ peak force (N)	Isokinetic dynamometer	Everything was given as needed	Greece
Thomas et al. [48]	10 (6/4)	64.7 ± 7.9	29.15 ± 2.5	Preoperative Six months postoperative	1,07 ± 1,45 1,28 ± 0,95	1,53 ± 1,26 1,50 ± 1,52	69,93	Max. isometric strength (Nm/kg)	Electromechanical dynamometer	WebPlotDigizier, SE in SD	United States
Topp et al. [49]	28 (18/8)	63.5 ± 6.68	32 ± 6.09	Preoperative Three months postoperative	54,02 ± 31,43 60,74 ± 25,45	82,74 ± 46,04 94,73 ± 46,62	65,29	Max. isokinetic muscle strength (torque/body weight)	Biodex System 3 Version 3.30 dynamometer	SE in SD	United States
Tsukada et al. [50]	27 (27/0)	74.1 ± 8.6	27.2 ± 4.6	Preoperative Three months postoperative	157,50 ± 57,75 156,62 ± 64,75	161,00 ± 42,88 186,38 ± 51,63	97,83	Isometric knee extension strength (N)	Microfet 2 Load Cell Dynamometer	WebPlotDigizier	Japan
Vahtrik et al. [52]	14 (14/0)	60.2 ± 7.6	34.7 ± 4.6	Preoperative Three months postoperative Six months postoperative	8,40 ± 3,37 7,10 ± 2,62 8,70 ± 4,49	11,10 ± 4,49 11,30 ± 4,86 13,70 ± 5,99	75,68	Maximal voluntary contraction (isometric):body mass	Custommade dynamometer	SE in SD	Estonia
Vahtrik et al. [52]	12 (12/0)	61 ± 6.8	33 ± 4.6	Preoperative Three months postoperative Six months postoperative	258,73 ± 60,48 150,00 ± 27,5 187,30 ± 52,24	322,20 ± 74,20 294,44 ± 71,46 317,46 ± 54,98	80,30	Max. isometric voluntary contraction (N)	Dynanometric chair equipment	WebPlotDigizier, SE in SD	Estonia
Vuorenmaa et al. [55]	43 (37/6)	70 ± 5	31 ± 5	Preoperative Three months postoperative	181 ± 35,71 138 ± 58,43	232 ± 29,22 236 ± 61,68	78,02	Max. Isometric knee strength (N)	David-200 dynamometer	Mean derived from change of first measurement; confidence interval in SD	Finland
Wada et al. [56]	20 (10/10)	70.8 ± 7.2	27.6 ± 2.2	Preoperative Six months postoperative One year postoperative	1,03 ± 0,44 1,15 ± 0,39 1,24 ± 0,34	1,35 ± 0,41 1,35 ± 0,41 1,29 ± 0,41	76,30	Peak isometric knee extionsion torque (Nm/kg)	Handhelt dynamometer	Everything was given as needed	Japan
Hopkins et al. [19]	19 (19/0)	66.1 ± 6.9	32.2 ± 7.1	Preoperative Six months postoperative One year postoperative	6398 ± 878 6173 ± 933 6138 ± 1148	6461 ± 888 6135 ± 768 6056 ± 858	99,02	Leg lean tissue mass (g)	Dual-energy X-ray absorptiometry (DXA) (GE Lunar Prodigy, Bedford MA)	Everything was given as needed	United Kingdom
Takamura et al. [46]	47 (39/8)	75.6 ± 7.7	27 ± 5.2	Preoperative Three months postoperative Six months postoperative	0,81 ± 0,31 0,66 ± 0,23 0,76 ± 0,2	1,01 ± 0,34 0,94 ± 0,29 0,94 ± 0,29	80,20	Nm/kg	Hand-held dynamometer (HHD; Anima, Tokyo, Japan)	Everything was given as needed	Japan
Ho et al. [18]	58 (46/12)	67.9	27.6	Preoperative Six months postoperative One year postoperative	12,56 ± 6,23 10,8 ± 4,99 15,53 ± 7,98	14,19 ± 7,61 14,07 ± 7,8 15,18 ± 8,36	88,51	Unknown	Dynamometer	Everything was given as needed	China
Şavkin et al. [39]	20 (1/19)	64.25 ± 5.52	31.85 ± 5.74	Preoperative 3 months postoperative	95,08 ± 22,9 98,01 ± 22,79	94,25 ± 17,3 100,9 ± 28,76	101,00	*N*	Hand-held dynamometer (Commander Muscle Tester, J Tech, USA)	Everything was given as needed	Belgium

Values are mean ± standard deviation

*BMI* Body Mass Index, *IQR* Interuqrtile range, *SD* Standard deviation, *SE* Standard error, *kg* kilogram, *N* Newton, *kgf* kilogram-force, *W* Watt, *Nm* Newton meters, *g* gram

### Risk of Bias of individual studies

#### Non-randomized studies

An overview of all non-randomized studies is presented in Figs. 2 and 3. All matched before and after studies except for one [55] displayed moderate to serious risks for a bias due to confounding factors and due to not containing information about what patients did before their operation. Merk et al. [29] study was the only study with serious bias due to selection of participants because no exclusion criteria were reported. The possibility that patients with other diseases took part in that study cannot be ruled out. Besides an anamnesis survey and a thigh circumference measurement were made but results were not reported. That is why bias due to missing data was classified with serious risk of bias and bias in selection of the reported result was classified moderate.

The surgery technique was not mentioned in four studies which led to a moderate risk of bias due to deviations from the intended intervention [18, 19, 29, 56]. Four studies did not specify the order of measuring of the examined parameters. These studies were judged with moderate risk of bias in measurement of the outcomes [3, 18, 29, 34].

#### Randomized controlled studies

The risk of bias for the randomized controlled trials are demonstrated in Figs. 4 and 5. Four studies [22, 39, 49, 50] were judged with high risk of bias arising from the randomization process (no allocation concealment). Five studies [22, 36, 39, 49, 50] contained no intention to treat analysis which led to a judgment of some concerns in domain two. Tsukada et al. [50] lost seven patients in follow-up measurements. Two of them were lost because of vein thrombosis. If thrombosis resulted from the operation, an exclusion of these two patients would distort reported results. Hence investigators assessed this study with some concerns for bias due to missing outcome data. Six studies were classified with some concerns in risk of bias in measurement of the outcome because assessment have been influenced by the knowledge of the outcome [20, 22, 30, 39, 49, 50].

### Result of the main syntheses: strength/torque

Meta-analysis for strength, neuromuscular activation and muscle mass were intended. Due to insufficient data, neuromuscular activation had to be excluded from this systematic review and muscle mass was only included in the systematic review but not in the meta-analyses.

#### Three months post-surgery

**Fig. 6:**
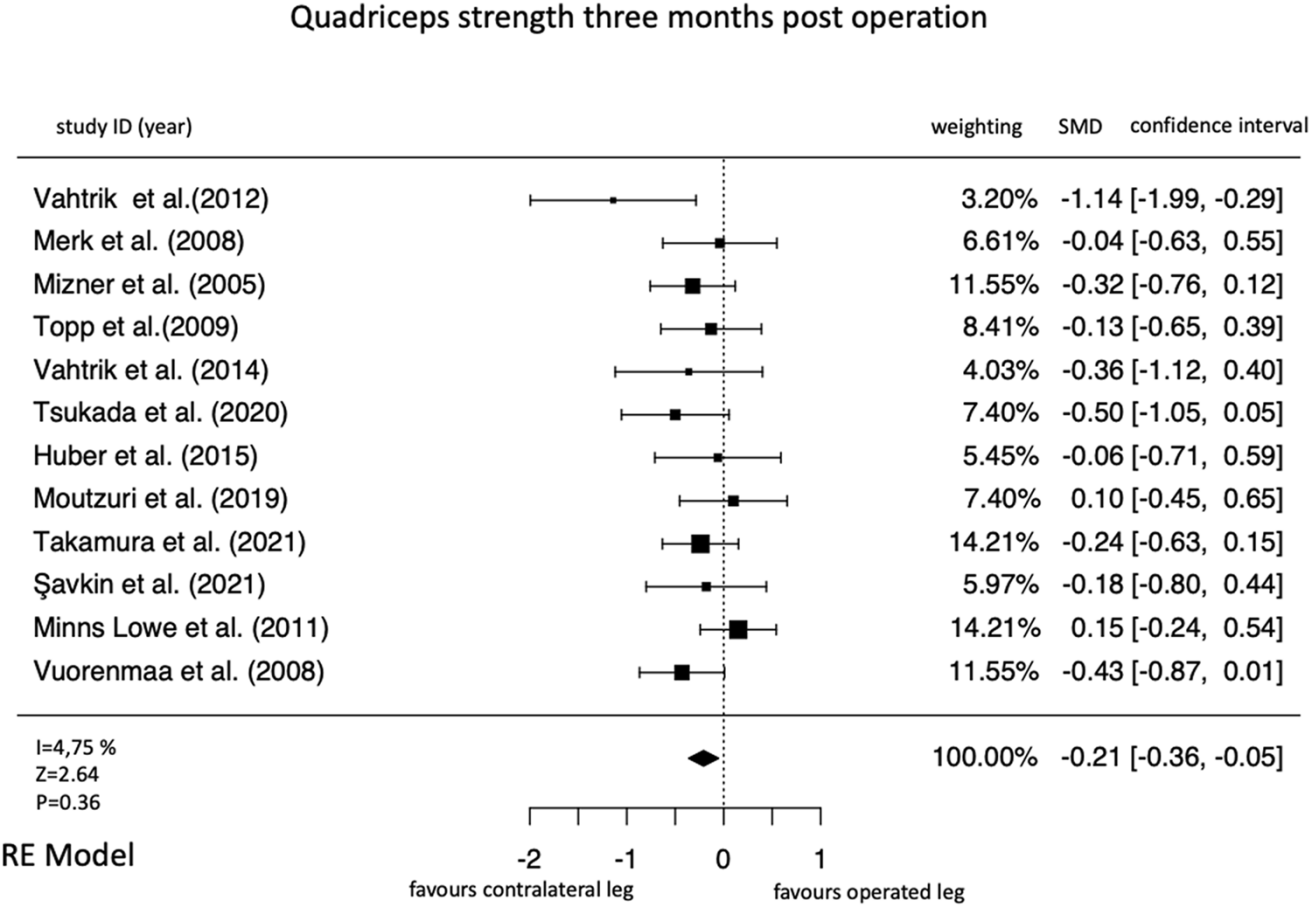
Forrest plot for the time point three months after the operation

At 3 months, the pooled effect size was negative (Fig. 6).

The overall quadriceps force-progression in the operated leg was significantly less when comparing it to the non-operated leg (Fig. 6). Since all studies reported stronger non-operated legs than operated legs preoperatively, findings from the meta-analyses indicate an increase in the contralateral differences. This result is significant (Fig. 6). The presented values for the strength progression from preoperation until 3 months after the operation have little heterogeneity (Fig. 6).

#### Six months post-surgery

**Fig. 7:**
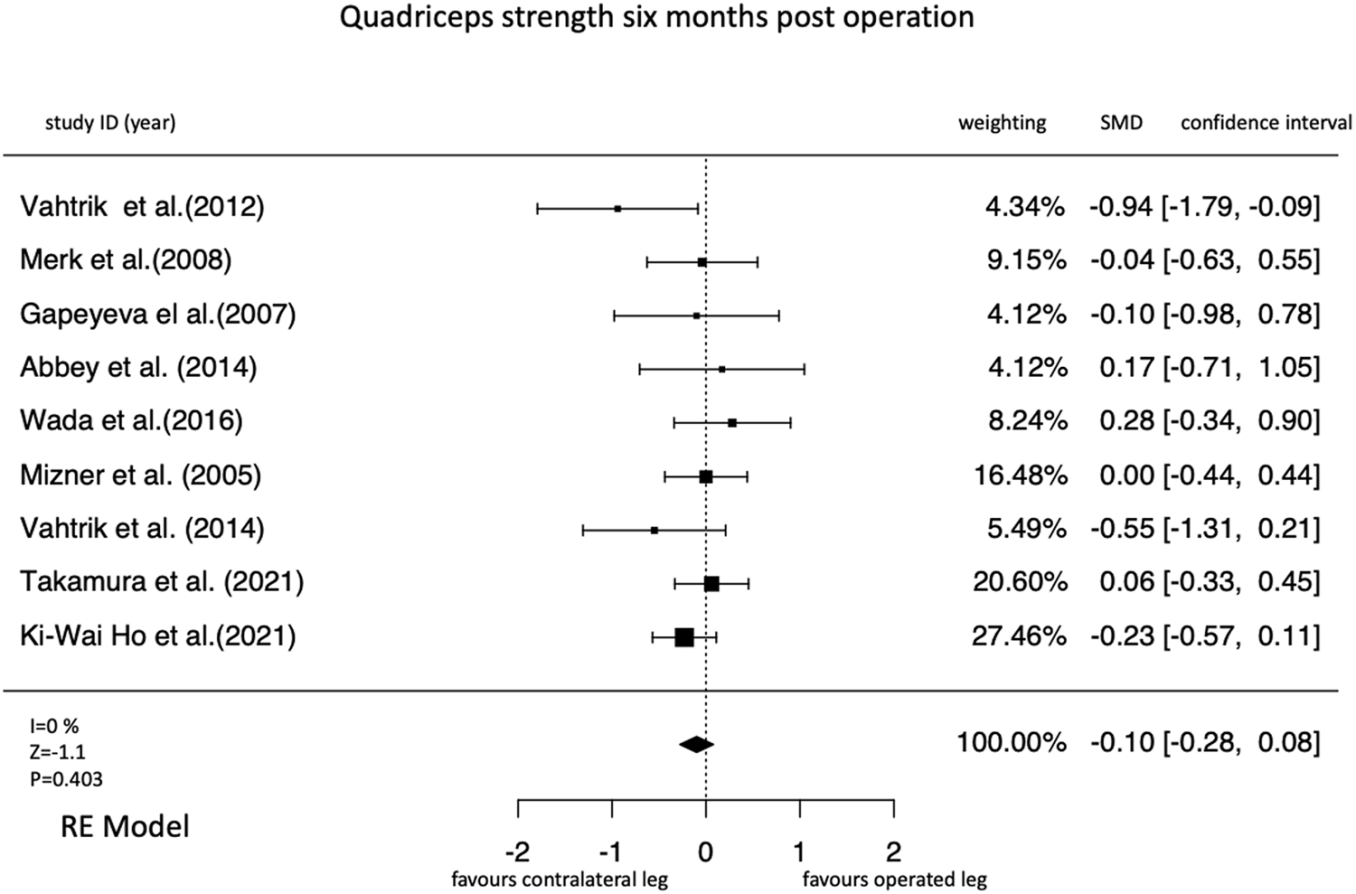
Forrest plot for the time point six months after the operation

At 6 months after the operation, no significant pooled effects could be reported (Fig. 7). Eight out of nine studies included in meta-analysis reported no significant outcomes. The presented values for the strength evolution from preoperation until 6 months after the operation have no heterogeneity (Fig. 7).

#### One year post-surgery

**Fig. 8:**
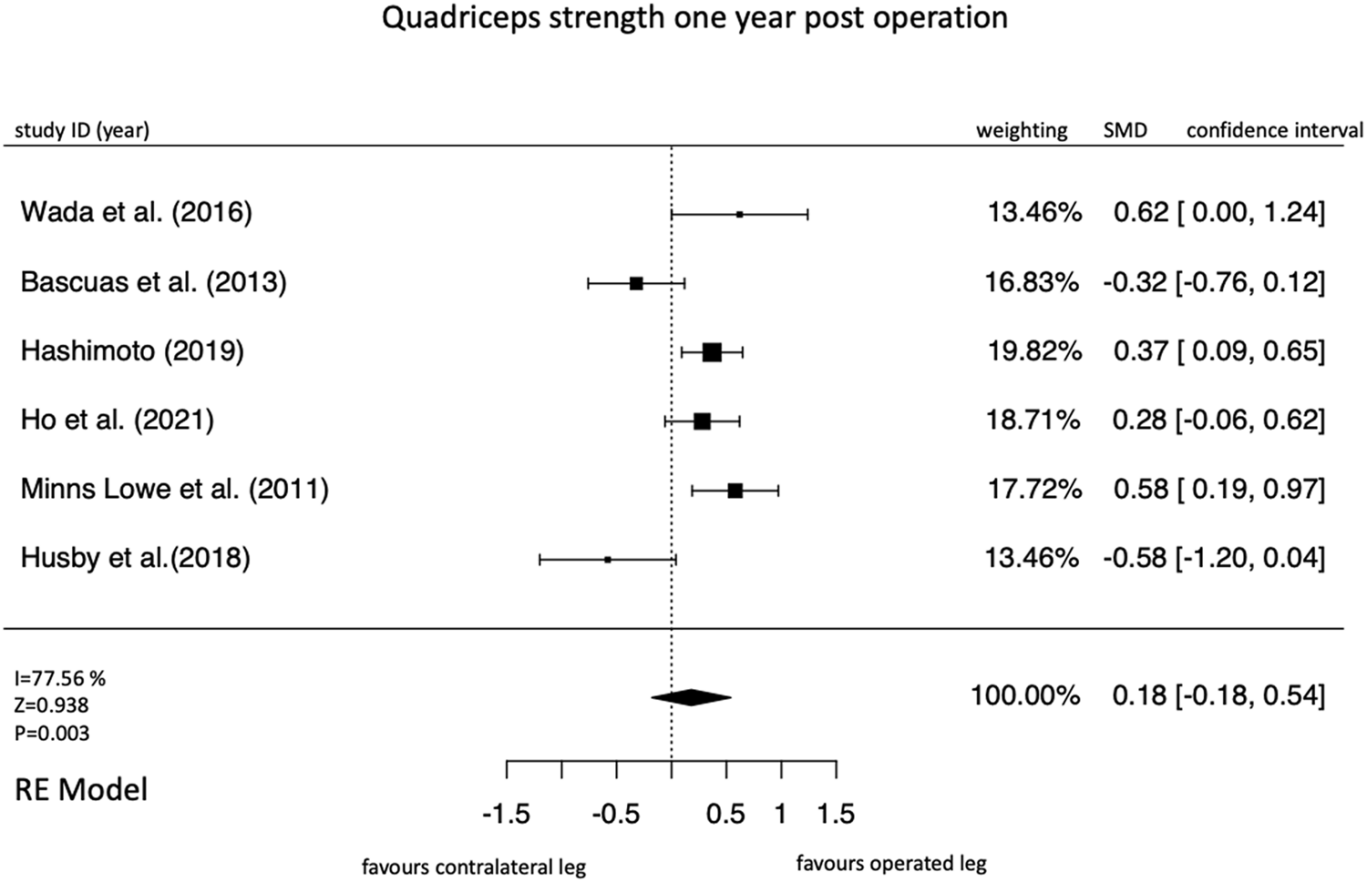
Forrest plot for the time point one year after the operation

One year after the surgery, the overall effect size was not significant (Fig. 8). The presented values for the force progression from pre-surgery to 1 year after surgery show a high heterogeneity (Fig. 8).

### Results of the lean leg tissue mass progression

In all studies, except for one [39], the operated leg was found to be weaker than the contralateral leg before surgery. 6 months and 1 year after the surgery, the operated leg was stronger than before the operation in most studies (Table 1). However, after surgery, the lean mass in both legs decreased [19]. The initial decrease in muscle mass from pre-surgery to 6 months after surgery was slowed, but lean mass continued to decrease from 6 months to 1 year after surgery in both legs [19].

### Meta-regression

All effect sizes except for one were included in the regression analysis. Moutzouris et al. [36] effect size was not included because the authors did not report information on the percentage of female participants and body mass index.

Results of the meta-regression are presented in Table 2. The variation in effect sizes can be attributed to a large extent to the examined independent variables (*R*^2^ = 0.31). No regressor significantly contributed to the effect size heterogeneity reduction.

**Table 2:** Meta-regression

Coefficients	Estimate	Standard error	Confidence interval lower level	Confidence interval upper level
Intercept	− 2.63	56,477	− 13,6992 84,396	84,396
Time after the operation (months)	0.0456	0.0495	− 0.0514	0.1427
Share of female participants (%)	− 0.0033	0.0073	− 0.0176	0.011
Age (years)	0.0299	0.0447	− 0.0577	0.1175
Body mass index (kg/m^2^)	0.0153	0.1041	− 0.1887	0.2192

*R*^2^ = 0.31; (cochranes) *Q* = 4.8

### Reporting Bias

**Fig. 9:**
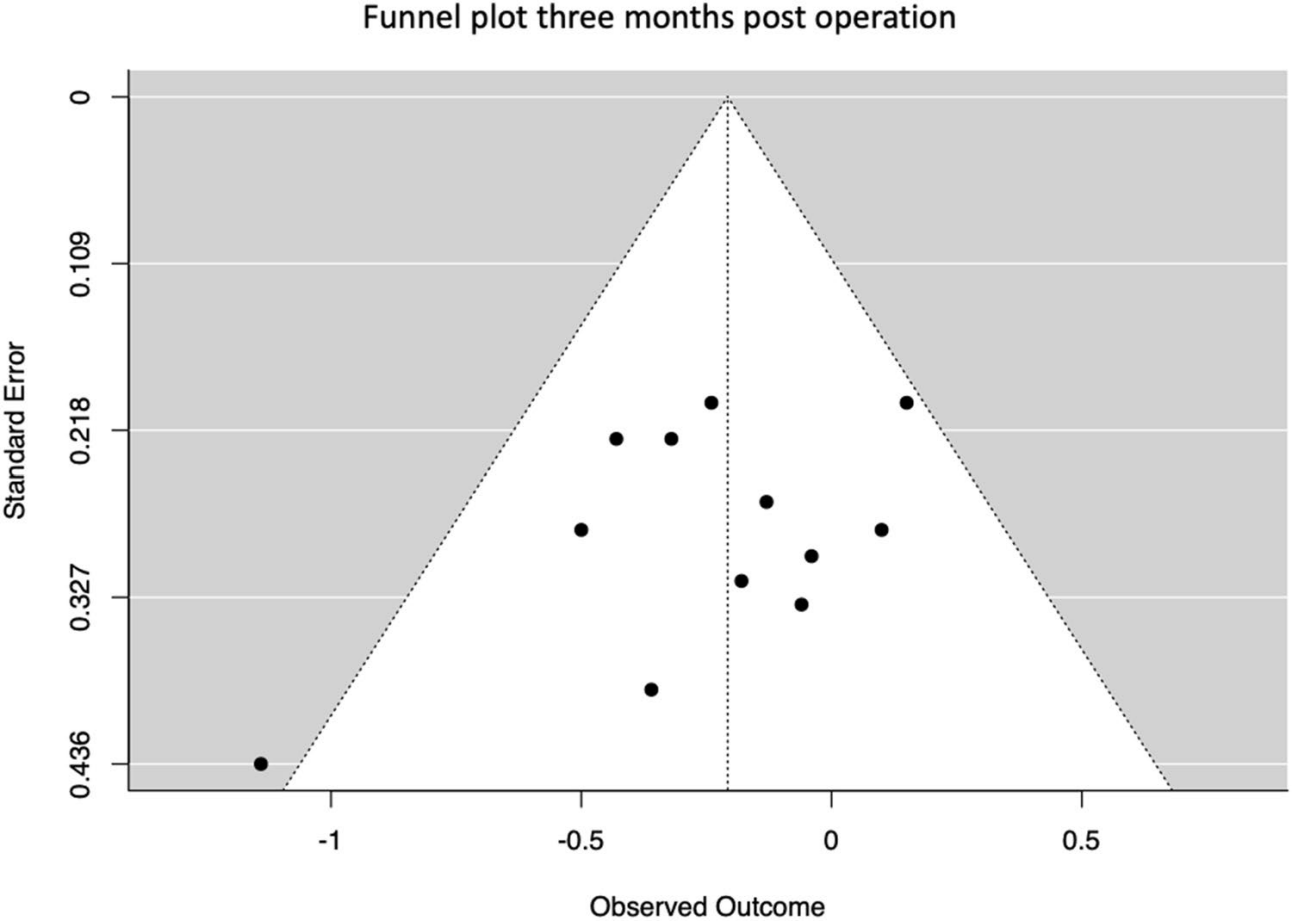
Funnel plot for the time point three months after the operation

Funnel plot (Fig. 9) and Egger’s Regression test (value = − 1312; *p* = 0.19) indicated no publication bias.

### Certainty of evidence

Since all studies were evaluated with Robins I and Rob 2 the initial classification was “high certainty” for all three meta-analyses. Due to a serious risk in the domains “Bias due to confounding” in studies evaluated with the Robins I and “bias arising from randomization process” in the Rob 2 tool, due to the high heterogeneity (downgrade, only 1 year after the surgery), due to the large confidence interval of the effect size at 1 year and due to the lack of publication bias assessment (downgrade, 6 months and 1-year post-surgery), the certainty of evidence was finally as follows: Meta-analysis for 3 and 6 months were classified to contain moderate quality of evidence and the meta-analysis for 1 year after the operation was classified to contain very low quality of evidence.

## Discussion

This systematic review with meta-analyses described and analyzed the course of skeletal muscle strength and mass of knee extensor muscles from preoperative to postoperative status until 1 year after a TKA intervention. We found very low certainty evidence that preoperatively existing imbalance of muscle strength and mass in favor of the leg not undergoing surgery is not counterbalanced within 1 year after primary TKA. Furthermore, the current results show, with moderate certainty evidence, that deficits in muscle strength of the knee extensors persist and progress until 3 months post-TKA persist in the operated leg until improvements can be detected.

In contrast to previously published meta-analyses, the present analyses only included studies that measured the operated leg and the contralateral leg as an intraindividual comparison. This approach was chosen because perceived pain scores and mobility in between-subject analyses are strongly affected by interindividual differences in sex, age, anthropometric characteristics, and activity level [25]. Therefore, present data are of significant practical importance for patients who are planning to undergo TKA and would like to know how their SMH is recovering during treatment.

As the preoperative muscle strength of the knee extensors, as well as the muscle mass and functional abilities of a patient, can be considered as positive predictive values for a successful rehabilitation, preoperative SMH has an important influence on the clinical outcome after TKA [8, 31]. As patient satisfaction after primary TKA is still only around 80% [5, 6], the results of our meta-analyses on the SMH of patients before and after TKA are most relevant for clinical care.

### Preoperative muscle mass and strength

All 20 included studies reported differences between the to-be-operated leg and the contralateral leg in favor of the contralateral one. The largest contralateral strength difference was reported by Merk et al. [29], where the operated-leg had only half of the strength of the contralateral one. Prior to the surgery, the average operated-leg had only one-third of the strength of the non-operated leg.

Degenerative joint diseases are associated with a decrease in the skeletal muscles mass of the affected limb [1, 41].

In addition to immobility-related atrophy, decreased neuronal activation also appears to contribute to the loss of muscle mass. For example, some data indicate that degenerative knee joint changes lead to decreased excitability of motoneurons and thus to decreased activation of the quadriceps, which is referred to as arthrogenic muscle inhibition [21]. Therefore, pain associated reductions in mobility and neuromuscular activations are key elements in preoperative existing muscle mass and strength reductions.

Since surgical therapy, implants and postoperative rehabilitation strategies have already been modified considerably, some studies have focused on increasing the physical capacities of patients preoperatively to increase clinical outcome. Following the maxim “Better In, Better Out”, a variety of so-called “prehabilitation protocols” were investigated to modify SMH already before surgery [45]. However, recent meta-analyses on prehabilitation show only a low to moderate impact on pre- and postoperative outcomes [37, 57]. Generally, the problem is that a degenerative joint is not able to perform the mechanical stimulus that is necessary for muscle and strength gain without causing increased pain. Therefore, new training methods are being investigated that are able to build up muscle without a high mechanical component and thus improve the preoperative and postoperative outcome of TKA [12].

### Postoperative rehabilitation of muscle mass and strength

Analysis of strength recovery after TKA-Surgery was done for 3-, 6 and 12 months postoperatively to demonstrate short- as well as long-term formal medically prescribed standard rehabilitation effects.

The results 3 months after TKA surgery showed that the total quadriceps force was significantly lower on the operated leg compared with the unoperated leg (Fig. 6). Since all studies reported stronger non-operated legs than operated legs preoperatively, present findings indicate an increase in the contralateral differences. These findings are well in line with the literature, describing the largest loss of muscle mass and muscle strength in the early phase of recovery after TKA [9, 11]. Since skeletal muscle tissue needs sufficient time to recover after traumatic events, whether of metabolic (e.g., muscle damage with atrophy induction due to ischemia/reperfusion injury through tourniquet use [27] or mechanical etiology (e.g., iatrogenic injury” [7], further progression after hospitalization could be caused by “immobility-induced atrophy” [16] and, “arthrogenic muscle inhibition after surgery” [32]. However, all studies that measured muscle strength at 3 and 6 months postoperatively reported enhancements for the operated leg [29, 30, 51, 52] (Table 1) which indicates that the surgery- and post-surgery-induced impairments are temporary and can be recovered. These findings are well in line with previous reported analysis, showing that postoperative muscle strength decreased 3 months after TKA and recovered to preoperative levels at 6 months post-surgery [35]. It must be noted that a negative effect size in the meta-analyses does not necessarily mean that the operated leg became weaker after surgery. It can also mean that the operated leg got stronger, but the strength increase was larger in the non-operated leg.

However, six out of seven studies included in our meta-analysis reported no significant outcomes for strength progression in comparison to the non-operated leg. As the overall effect size of the meta-analysis is slightly negative the preoperative contralateral differences cannot be balanced, it seems more that this disparity is increasing (Fig. 7). The results of long-term rehabilitation show that the operated leg becomes increasingly stronger, but the existing imbalance to the non-operated leg also increases up to 1 year after surgery (Table 1).

Several studies compared postoperative rehabilitation of muscle mass and muscle strength after TKA with age-matched healthy control patients. The results show that quadriceps strength still reaches only 70–80% of the strength of healthy controls 1 year after TKA [4]. Furthermore, results by Schache et al. [40] revealed that muscular weakness is continuously evident up to 3 years after surgery in comparison to healthy controls. The authors concluded that improving muscle strength postoperatively to a level similar to that of healthy control participants could improve patient dissatisfaction after TKA. However, our data show that 1 year after TKA, most patients are not even able to reach a level of muscle strength that is close to their non-operated leg. A comprehensive comparison of the operated to the non-operated leg has so far been considered as a research desideratum due to the limited data available [40]. Consequently, with appreciation of our data, it becomes apparent that the objective of rehabilitation interventions should be to match the muscle strength of both legs in the first place.

In addition to aforementioned results, present analyses demonstrate that certain physical characteristics have a negative influence on regeneration after TKA (Table 2). Studies with a higher percentage of female patients report a poorer strength progression of the operated leg compared to the non-operated leg (Table 2). These findings stay in contrast to research articles, describing a faster improvement in WOMAC score for woman after primary TKA [26]. However, our results suggest that standard care (surgery + standard rehabilitation) does not have a significant impact on muscular strength of the operated leg of women. Therefore, a special training program which focuses on muscle strength, hypertrophy and neuromuscular activation for women could be a useful postoperative tool to increase muscle strength and satisfaction after primary TKA.

Additionally, a negative correlation between higher BMI and strength progression after the surgery was found (Table 2). A higher BMI led to a lower strength progression of the operated leg compared to the contralateral leg. In fact, overweight patients tend to build up less muscular strength in the operated leg after TKA [38]. However, a recent review by Godziuk et al. [15] concluded that there is currently no evidence to support the benefit of preoperative weight loss on postoperative outcomes after TKA. Due to the lack of representative research, preoperative weight loss is further an individual therapeutical step between surgeon and patient. Nevertheless, based on our data, it appears that an increased BMI has a negative effect on muscular regeneration. Therefore, special pre- or postoperative treatment protocols with the aim to reduce weight and strengthen the muscles of the lower extremities could be a promising tool to enhance SMH rehabilitation after TKA.

In the comparison 1 year after TKA, it is particularly interesting that the confidence intervals of the effect sizes of the meta-analyses do not overlap, in contrast to the other two time points (Fig. 8). This indicates that patients’ strength progression in the studies is very different and thus the confidence interval of the overall result is very large (pooled effect size = 0.15; CI 95% − 0.30, 0.61) (Fig. 8). However, due to the positive overall effect size, it can be verified that after 1-year post-TKA the differences between the legs become smaller and the strength of the operated leg improves (Fig. 8).

### Methodical procedure

The present results are based on reported outcomes from 20 included studies. As examination of the contralateral leg is performed infrequently, little information on differences between the operated and non-operated leg is available. Additionally, only six studies could be included for comparison after 1 year, which implies that no publication bias assessment could be done because of a lack of studies. These results show a significant research desideratum and highlight the importance for more studies including the contralateral leg in TKA, as well as longer study periods to monitor muscular regeneration.

Furthermore, difficulties were experienced in collecting the data for calculating the meta-analyses. Whereas some studies reported only the standard error, confidence interval or the interquartile range instead of reporting mean and standard deviations. Other studies reported the SD in the form of bar charts, which indicate that necessary standard deviation was extracted by software tools, here Web Plot Digitizer (Table 1).

In the interpretation of the results, another problem resulted from the fact that many studies showed an extensive wide confidence interval (Table 1). The explanation of this relates to the very individual rehabilitation of the patients included. The pattern of rehabilitation seems to be not linear, while some rehabilitate quickly, others still face long-term problems. In future studies, daily physical activity of the patients has to be considered to investigate differences in rehabilitation after TKA.

Although it is known that preoperative SMH is important for rehabilitation after TKA only one study reported preoperative trends [16] (Fig. 2). Preoperative fitness level seems to have a significant impact on rehabilitation quality. We suggest that preoperative exercise therapy could have a unique impact on recovery after surgery and postoperative activity. However, this has also not been investigated and should be focused in future studies.

The irregularities presented in the results of this systematic review can be explained by the fact that after standard rehabilitation the amount of exercise was not standardized anymore. Patients who were physically more active obviously gained more strength. Additionally, the two influencing variables BMI and female gender have a major impact on muscular regeneration after surgery.

### Conclusion and relevance for practice

In conclusion, present data suggest that strength progression after primary TKA is not consistent and linear. Although on average the operated-leg got stronger from 3 months after the operation onwards, no significant improvement of the operated-leg strength compared to the non-operated leg was found, until 1 year after TKA. Since one of the postoperative goals of this surgical intervention is to rebuild muscular function, muscular strength and especially the treatment of existing strength imbalances, standard rehabilitative care does not seem to be sufficient.

Future studies should try to focus on how preoperative imbalances affect postoperative outcomes and which interventions can be applied pre- as well as postoperatively to reduce existing imbalances.

Considering, that SMH is currently only a secondary therapy goal of a TKA intervention, beyond pain reduction and restoration of physiological mobility, its impact on postoperative outcome can be counted as significant. Therefore, pre- and postoperative improvements in SMH could be a successful tool to enhance clinical outcomes and patient satisfaction after TKA.

## Data Availability

The data that support the findings of this study are available from the corresponding author upon reasonable request. Source data underlying all Figures and Tables are provided as a Source.
