# Correction to: The course of knee extensor strength after total knee arthroplasty: a systematic review with meta‑analysis and ‑regression

**DOI:** 10.1007/s00402-023-04808-y

**Published:** 2023-02-21

**Authors:** Ravi Singla, Daniel Niederer, Alexander Franz, Kevin Happ, Christoph Zilkens, Patrick Wahl, Michael Behringer

**Affiliations:** 1 Department of Orthopedics and Trauma Surgery, University Hospital Bonn, Bonn, Germany; 2 Department of Adult Reconstruction, ATOS Orthoparc Clinic Cologne, Cologne, Germany; 3 Institute of Cardiology and Sports Medicine, German Sport University Cologne, Cologne, Germany; 4 Department of Sports Medicine and Exercise Physiology, Institute of Occupational, Social and Environmental Medicine, Goethe University Frankfurt, Frankfurt, Germany; 5 Department of Sports Sciences, Goethe University Frankfurt, Frankfurt, Germany

**Correction to: Archives of Orthopaedic and Trauma Surgery** 10.1007/s00402-022-04750-5

The original version of this article unfortunately contained a mistake. Author entered a wrong country in Table 1. The study by Şavkin et al. (2021) (reference #39) was not conducted in Belgium, as stated in the article, but in Türkiye.

